# N-terminal Pro-B-Type Natriuretic Peptide and Malnutrition in Patients on Hemodialysis

**DOI:** 10.1155/2020/9528014

**Published:** 2020-03-05

**Authors:** Jacques Ducros, Laurent Larifla, Henri Merault, Valérie Galantine, Valérie Bassien-Capsa, Lydia Foucan

**Affiliations:** ^1^Service de Néphrologie, Centre Hospitalier Universitaire, Guadeloupe, France; ^2^Service de Cardiologie, Centre Hospitalier Universitaire, Guadeloupe, France; ^3^Equipe de recherche Epidémiologie Clinique et Médecine sur le Risque Cardio métabolique, ECM/LAMIA, EA 4540, Université des Antilles, Centre Hospitalier Universitaire, Guadeloupe, France

## Abstract

Natriuretic peptides, brain natriuretic peptide (BNP), and N-terminal probrain natriuretic peptide (NT-proBNP) are mainly known as diagnostic markers for heart failure with high diagnostic and prognostic values in the general population. In patients who are undergoing hemodialysis (HD), changes in NT-proBNP can be related to noncardiac problems such as fluid overload, inflammation, or malnutrition and can also be influenced by the dialysis characteristics. The current review aimed to summarize findings from studies on the association between NT-proBNP and malnutrition in HD patients. Articles published after 2009 and over a ten-year period were considered for inclusion. We first briefly discuss the traditional functions of NT-proBNP, and after, we describe the functions of this prohormone by focusing on its relation with protein energy wasting (PEW) in HD patients. Mechanisms that could explain these relationships were also discussed. Overall, 7 studies in which the investigation of the relations between NT-proBNP and nutritional status in HD patients were among the main objects were taken into account. NT-proBNP levels correlated with several factors described in the 4 categories of markers indicative of PEW (body mass and composition, muscle mass, biochemical criteria, and dietary intakes) and/or were associated with PEW. Interactions between several parameters could be involved in the association between NT-proBNP and malnutrition with a strong role of weight status. NT-proBNP is elevated in HD patients and is associated with malnutrition. Nevertheless, the prognostic value of NT-proBNP on nutritional status should be evaluated.

## 1. Introduction

Uremic malnutrition, also called malnutrition inflammation complex syndrome or protein energy wasting, corresponding to a decrease in energy and body protein, is a common problem in patients with end-stage renal disease (ESRD) undergoing HD and has been consistently associated with mortality in different populations [1–5].

Brain natriuretic peptide (BNP) is synthesized mainly in the heart as a proBNP that is further cleaved into bioactive BNP and biologically inactive NT-proBNP [6]. Both BNP and NT-proBNP are released in response to changes in pressure inside the heart that are related to heart failure and other cardiac problems. Thus, BNP and NT-proBNP are mainly known as cardiac biomarkers with high diagnostic and prognostic values [7–9] with widespread use in cardiac diseases [10]. In HD patients, changes in NT-proBNP can be related to noncardiac problems such as fluid overload, inflammation, or malnutrition. In this line, NT-proBNP was recently associated with 2-year mortality from both cardiovascular and noncardiovascular origins in 1,310 prevalent chronic HD patients [3].

The current review aimed to summarize findings from studies on the association between NT-proBNP and malnutrition in HD patients.

## 2. Methods

For this review, we reviewed the literature using PubMed, Medline, and Scopus with the following search terms: N-terminal probrain natriuretic peptide (NT-proBNP), nutritional status, malnutrition, and hemodialysis (HD) in humans. We also searched in the selected articles and took into account some of the relevant references cited by the authors. Mechanisms that could explain these relationships were also discussed while also taking the results of some animal studies into account.

Articles published after 2009 and over a ten-year period, in adults (>18 year of age), were considered for inclusion.

We first briefly discuss the characteristics and traditional functions of NT-proBNP, and after, we describe the functions of this prohormone by focusing on its relation with protein energy wasting (PEW) in patients undergoing maintenance HD.

## 3. Results

During the period taken into account in this review, we found 7 studies in which the investigation of the relations between NT-proBNP and nutritional status in HD patients were among the main objects [11–17]. These studies are summarized in Table 1.

## 4. Discussion

### 4.1. NT-proBNP Levels in HD Patients

The levels of the prohormone vary according to the population studied. NT-proBNP is cleared by the kidney and, in patients with CKD, especially those on HD, levels of NT-proBNP are usually increased as a consequence of increasing secretion and decreasing renal clearance [17].

Thus, elevated levels of the prohormone are often observed in HD patients without clinical evidence of cardiovascular disease. These high NT-proBNP levels in patients with renal dysfunction [11, 12, 18–20] were higher than in individuals with normal renal function.

### 4.2. Factors Other than Cardiac Status That Influence NT-proBNP Levels in HD Patients

- The influences of age, gender, body mass index (BMI), time of the dialysis session during the week, time of measurement of NT-proBNP before or after a hemodialysis session, dialysis characteristics, and membrane type have been reported [21–24].
- In some studies, blood concentrations of NT-ProBNP were associated with hypervolemia or fluid overload [11, 25–28]. Results of bioimpedance monitoring, a tool that defines the influence of fluid overload in body mass index and clinical dry weight assessment, can also be affected by malnutrition with loss of cell mass [26].
- Inflammation also influences NT-proBNP levels [13, 17, 29].

### 4.3. Assessment of Malnutrition

Studies on maintenance of HD patients have reported the association between NT-proBNP levels and malnutrition by subjective global assessment (SGA), malnutrition-inflammation score (MIS), or markers defined in the International Society of Renal Nutrition and Metabolism (ISRNM) nomenclature [11–17].

Globally, SGA is a tool that uses 5 components of a medical history (weight change, dietary intake, gastrointestinal symptoms, functional capacity, and disease and its relation to nutritional requirements) and 3 components of a brief physical examination (signs of fat and muscle wasting, nutrition-associated alterations in fluid balance) to assess nutritional status [30]. The MIS has 10 components, each with four levels of severity ranging from 0 (normal) to 3 (very severe). The sum of all components ranges from 0 to 30. Higher scores reflect more severe malnutrition and inflammation [31].

The practice guidelines and criteria for evaluating nutritional status in ESRD patients recommend the use of nomenclatures for PEW [32, 33]. In the nomenclature, proposed by the ISRNM [32], several parameters among four established categories (body mass and composition, muscle mass, biochemical criteria, and dietary intakes) are indicative of PEW in individuals with kidney disease. At least 3 out of the 4 listed categories must be satisfied for the diagnosis of kidney disease-related PEW.

### 4.4. Relations between NT-ProBNP and Nutritional Status

To study these relationships, we first focused on the relation between the main parameters described in the 4 categories of factors for PEW identification according to the ISRNM nomenclature, and after, we considered the relation with malnutrition status in each of the studies.

#### 4.4.1. NT-proBNP and Body Mass and Composition

Lower BMI, body fat, and unintentional weight loss over time are included in this category of nutritional markers in the ISRNM nomenclature.

*(1) Body Weight-BMI*. Inverse relationships between BMI and circulating levels of NT-proBNP have been demonstrated, and lower BMI has been associated with higher NT-proBNP levels in subjects with and without heart failure [25, 34–38] as in patients with ESRD undergoing HD [11–16, 27, 39].

*(2) Weight Loss*. Weight loss is known to increase natriuretic peptide levels [40–43]. In HD patients, simple correlations between log NT-proBNP and percent weight loss have been found [26]. NT-proBNP levels were also associated with unintentional weight loss (−5% over 3 months) in another sample of HD patients [12], and positive correlation between NT-proBNP and IDWG was also reported [11].

*(3) Body Fat*. In HD patients, fat tissue index decreased across NT-proBNP quartiles [16], and lower total fat mass was found in those with greater NT-proBNP values [14]. These authors also investigated NT-proBNP and lean body mass (LBM) at baseline and at 12 months. LBM over 12 months was significantly decreased above the threshold of log NT-proBNP with 8.5 being the equivalent of 5,000 pg/mL [14].

#### 4.4.2. NT-proBNP and Muscle Mass

Assessment of nutritional status and especially evaluation of muscle mass is essential for the identification of patients at risk for the development of PEW [44]. Indirect measures of muscle mass such as serum creatinine (before a HD treatment), creatinine appearance, creatinine generation rate (%CGR), and creatinine index have been proposed. High NT-proBNP has been associated with decreased levels of creatinine, decreased creatinine index, or % CGR in HD patients [12–14].

#### 4.4.3. NT-proBNP and Biochemical Criteria

Some biochemical indicators have been proposed for the diagnosis of PEW, and serum albumin, serum prealbumin (transthyretin), and cholesterol have been studied as nutritional markers in CKD patients [32]. Negative relationships were found between serum albumin and NT-proBNP levels by some authors [11, 13, 16, 26] and not by some others [14]. But low serum albumin concentration is not sufficient for the diagnosis of PEW, although it is often present in this condition [32]. Natriuretic peptides also affect lipid metabolism and plasma cholesterol [16, 45].

#### 4.4.4. NT-proBNP and Dietary Intakes

In the ISRNM nomenclature, low dietary intake was considered in the presence of unintentional low dietary protein intake <0.8 g/kg/day for at least 2 months for dialysis patients or unintentional low dietary energy intake <25 kcal/kg/day for at least 2 months [32]. The normalized protein catabolic rate (nPCR) has been proposed as a useful measure for dietary protein intake and ultimately for nutrition. The HD procedure can enhance protein catabolism owing to dialytic losses of protein and amino acids and to the inflammatory response to blood-dialyzer interaction [46, 47]. The nPCR is a parameter of intensive catabolism. In HD patients with intensive catabolism, nPCR was one of the 4 parameters influencing serum NT-proBNP [11]. Simple negative correlation between log NT-proBNP and nPCR was also found in another study [12], but no relation was found by other authors [14].

#### 4.4.5. NT-proBNP and Protein Energy Wasting

The 7 studies in which the investigation of the relations between NT-proBNP and nutritional status in HD patients were among the main objects are summarized in Table 1.

In 222 HD patients, the association between NT-proBNP, PEW, and inflammation was evaluated. NT-proBNP was analyzed in plasma by an immunometric assay. Patients presenting with an SGA score of 2–4 were defined as malnourished. A NT-proBNP level above 9,761 pg/ml was associated with PEW even following adjustment for age, dialysis vintage, inflammation, and Davies score [13].

In 97 HD patients, the relationship between serum level of NT-proBNP and nutritional status, inflammation, and hydration was investigated. NT-proBNP was measured by an immunoassay. The nutritional status was evaluated with specific markers including BMI, albumin, and nPCR. NT-proBNP was only moderately associated directly with hydration status but was extremely elevated in patients with intensive catabolism [11].

In 44 HD patients, nutritional status was assessed using subjective SGA and MIS. NT-proBNP was measured by immunoassay, malnutrition was accompanied by volume overload and associated with increased log NT-proBNP, and these levels were independently associated with increased left ventricular mass index [15]. The authors suggested a possibility that nutritional status may affect ventricular remodeling in hemodialysis patients [15].

A prospective study was performed in 211 HD patients to analyze NT-proBNP variability and the factors predicting this variability. Malnutrition was one of the studied factors. NT-proBNP was measured by an immunometric assay. Longitudinal changes in NT-proBNP were associated with changes in nutritional status. Patients with wasting and patients with congestive heart failure had significantly higher NT-proBNP levels than patients without these conditions [17].

A study aiming to establish the usefulness of NT-proBNP for hydration assessment and the relation of NT-proBNP with the nutritional state and prognosis of survival was conducted in 321 HD patients. NT-proBNP was measured by immunoassay. Bioimpedance analysis was used. Patients were classified according to quartiles of NT-proBNP. NT-proBNP correlated negatively with practically all nutritional indices including serum albumin, cholesterol, and BMI. The highest albumin level was present in Q1 (4.10 ± 0.63/3.99 ± 0.51/3.90 ± 0.62/3.97 ± 0.78 g/dl; *P* = 0.006). The TC level was the lowest in Q4 (190 ± 60/169 ± 56/173 ± 51/153 ± 56 mg/dl; *P* = 0.002). But, although the data showed a relation of NT-proBNP with nutritional status, this link was less conspicuous than the link between NT-proBNP and hydratation [16].

In a prospective observational study with one-year follow-up in a cohort of prevalent HD patients (*n* = 238), NT-proBNP levels were measured by fully automated electrochemiluminescence. Nutritional status and changes in muscle mass were evaluated by subjective global assessment, %CGR, and creatinine index. NT-proBNP was significantly higher in HD patients with PEW. Prevalent HD patients with higher NT-proBNP (NT-proBNP ≧5,760 pg/mL) showed a high prevalence of malnutrition (30% vs 11% for those with lower NT-proBNP levels, *P* = 0.0002), decreased levels of creatinine, and decreased creatinine index. The authors suggested that compared with serum albumin, measurement of NT-proBNP could be a superior predictor of muscle loss in patients on HD. Patients with the highest NT-proBNP also showed a high prevalence of cardiac events and malnutrition [14].

The association between PEW and NT-proBNP was evaluated in a cross-sectional study performed in 207 Afro-Caribbean HD patients. One component in each of the 4 categories for the wasting syndrome (according to the ISRNM nomenclature) was retained: serum albumin <38 g/L, BMI < 23 kg/m^2^, serum creatinine < 818 *μ*mol/L (for this sample of Afro-Carribean HD patients), and nPCR < 0.8 g/kg/day. NT-proBNP was assessed using a chemiluminescence immunoassay. The frequency of individuals with low serum albumin level was higher in those with NT-proBNP ≥6,243 pg/mL than in the others (59.6% vs 43.2%, *P* = 0.041). A negative correlation between log NT-proBNP and nPCR was also found (*r* = −0.15; *P* = 0.028). High levels of NT-proBNP (≥6,243 pg/mL) were independently associated with PEW. NT-proBNP concentrations increased with PEW component number (on the basis of 1 in each of the 4 listed categories of PEW markers) [12].

In some other studies, authors found associations between NT-proBNP and signs of wasting, but the investigation of these relations was not the main objects in these studies [26, 48].

### 4.5. Hypotheses to Explain the Relation between NT-proBNP and Protein Energy Wasting

Several hypotheses have been proposed concerning the association between high NT-proBNP and PEW.

- A direct effect of PEW on the level of NT-proBNP by affecting ventricular remodeling in HD patients has been suggested [15].
- Complex interactions between NT-proBNP, malnutrition, inflammation, and fluid overload have been reported in HD patients [17, 49, 50]. In addition, bioimpedance results affected by fluid overload can also be affected by malnutrition
- Several parameters among 4 established categories (body mass and composition, muscle mass, biochemical criteria, and dietary intakes) for the definition of PEW according to ISRNM are separately associated with NT-proBNP. Interaction between some of these parameters could be involved in the association between NT-proBNP and malnutrition with a strong role of weight status [12]. In fact, HD patients with greater protein and energy intakes usually have a greater BMI [5] and the inverse. Hypoalbuminemia is the result of the combined effects of inflammation and inadequate protein and caloric intake [51] that could lead to low BMI. Creatinine level is a surrogate of muscle mass in HD patients [52]. Anorexia and low nutrient intake could also lead to lower muscle mass, lower creatinine levels, and lower BMI.
- Other hypothesis, suggested in patients without CKD, might be involved in HD patients.

Adipose tissue could also play an important role. It has been shown that NP display a lipolytic effect in adipose tissue [53, 54] and enhance the oxidative capacity of human skeletal muscle [55]. A heart adipose tissue connection has been suggested [56] as well as a link between NT-proBNP, PEW, and the appetite-modulating hormone ghrelin. Ghrelin and acyl ghrelin levels are associated with nutritional markers in HD patients [57], and low acyl ghrelin level was found associated with high NT-proBNP in male HD patients [57]. Plasma des-acyl ghrelin levels were significantly higher in HD patients than in controls and in anorexic HD patients than in nonanorexic [58] suggesting that des-acyl ghrelin, which induces a negative energy balance, could be involved in the complex pathogenesis of anorexia that is frequently found in HD patients [58, 59].

## 5. Conclusions

In summary, the data suggest that NT-proBNP correlates with the indices of protein energy wasting and malnutrition. Therefore, this marker seems to be useful in the evaluation of nutritional status in hemodialysis. However, limitations in most of the above studies include the small sample size, the isolated measurements of the biomarker, the lack of precision regarding the time of NT-proBNP measurement in some studies, and the potential biases related to the methods used for PEW assessment. Factors indicating the presence of PEW can also be induced by inflammatory processes. Thus, the clinical context should be taken into account for the interpretation of elevated NT-proBNP levels. Since high NT-proBNP is primarily a marker of cardiac dysfunction, the increased levels of the prohormone must draw attention to cardiac function but also to nutritional status. Nevertheless, we must highlight the need to evaluate the prognostic value of NT-proBNP for malnutrition in hemodialysis in further studies.

## Figures and Tables

**Table 1:** Summary of studies on relationships between NT-proBNP and protein energy wasting in maintenance hemodialysis.

Authors	Country	Number of HD subjects	Study design Assessment of malnutrition Men/women Age (years)	NT-proBNP levels
Guo et al. [13]	Sweden	**222**	Cross-sectional study and longitudinal study SGA 120/102 59 (47–72) y (median (IQR))	Values (median (IQR)): 11,609 (4,581–35,000) with wasting signs (SGA > 1) 5,671 (1,909–17,141) pg/ml in those without Negatively associated with nutrition markers, including serum albumin, IGF-1, handgrip strength, serum creatinine, and body weight Higher values (>9,761 pg/ml) associated with PEW (SGA > 1) Independently predicted PEW Nonsurvival had a poorer nutritional status

Bednarek-Skublewska et al. [11]	Poland	**97**	Cross-sectional study Indices of nutritional status and BIA 57/40 20–92 y (range)	Values (range): 403–35,000 pg/ml Negatively correlated with BMI, albumin, and transferrin Elevated in patients with intensive catabolism

Lee et al. [15]	Korea	**44**	Cross-sectional study SGA and MIS 21/23 53.9 ± 9.2 y (mean ± SD)	Values (median (IQR)): 4,342 (1,582–22,304) pg/ml in well-nourished 24,807 (11,435–44,127) pg/ml in malnourished Negatively correlated with fat mass Positively correlated with MIS Elevated in those with malnutrition

Snaedal et al. [17]	Sweden	**211**	Longitudinal study SGA 55/156 66 (51–74) y (median (IQR))	Values (median (IQR)): 8,946 (2,909–26,571) pg/mL 12,932 (5,658–35,001) pg/mL with PEW (SGA > 1) 6,092 (2,248–17,670) pg/mL without PEW Associated with malnutrition (SGA > 1) Changes in NT-proBNP associated with change in nutritional status Worse nutritional status was significantly related to increased variability

Schwermer et al. [16]	Poland Sweden	**321**	Longitudinal study Indices of nutritional status and BIA 206/115 65 ± 21 y (mean ± SD)	Values: (mean ± SD): 6,098 ± 19,659 NT-proBNP correlated negatively with practically all nutritional indices Cohort divided into NT-proBNP quartiles (with lowest values in Q1) BMI and fat tissue index decreased across NT-proBNP quartiles; highest albumin level was present in Q1 Lowest total cholesterol level in Q4

Ikeda et al. [14]	Japan	**238**	Longitudinal study SGA 149/89 64 ± 13 y (mean ± SD)	Values (median (range)): 2,910 (465–78,400) pg/ml Associated with muscle loss in malnourished HD patients Independently predicted the decreased change of LBM and the index of muscle loss Higher NT-proBNP levels: Decreased levels of creatinine, creatinine index as well as %CGR, lower total fat mass, and LBM and higher frequency of malnutrition

Ducros et al. [12]	Guadeloupe (France)	**207**	Cross-sectional study ISRNM nomenclature for PEW 112/95 64 ± 13 y (mean ± SD)	Values (range): 125–33,144 pg/ml (median (IQR)): 6,243 (1833–18,721) pg/mL with PEW 2,132 (1,100–5,200) pg/mL without PEW Elevated in those with BMI ≤23 Kg/m2), albumin ≤38 g/L, creatinine, ≤818 *μ*mol/L, nPCR ≤0.8 g/kg/d) and PEW (at least 3 among the 4 parameters) Negatively correlated with BMI and nPCR Increased with the increasing of the PEW marker number Values ≥6243 pg/mL independently associated with PEW

BIA: bioimpedance analysis. %CGR: percentage creatinine generation rate. MIS: malnutrition-inflammation score. IQR: interquartile range. ISRNM: International Society of Renal Nutrition and Metabolism. PEW: protein energy wasting. SD: standard deviation. SGA; subjective global assessment.

## Acronyms

BIA: Bioimpedance analysis
BMI: Body mass index
BNP: Brain natriuretic peptide
CKD: Chronic kidney disease
ESRD: End-stage renal disease
HD: Hemodialysis
hsCRP: High-sensitive C-reactive protein
IDGW: Interdialytic weight gain
ISRNM: International Society of Renal Nutrition and Metabolism
MIS: Malnutrition-inflammation score
NP: Natriuretic peptides
nPCR: Normalized protein catabolic rate
NT-proBNP: N-terminal probrain natriuretic peptide
%CGR: Percentage creatinine generation rate
PEW: Protein energy wasting
SGA: Subjective global assessment.
